# Identification and Expression Profile of CYPome in Perennial Ryegrass and Tall Fescue in Response to Temperature Stress

**DOI:** 10.3389/fpls.2017.01519

**Published:** 2017-11-20

**Authors:** Xiang Tao, Ming-Xiu Wang, Ya Dai, Yan Wang, Yan-Fen Fan, Ping Mao, Xin-Rong Ma

**Affiliations:** ^1^Chengdu Institute of Biology, Chinese Academy of Sciences, Chengdu, China; ^2^College of Life Sciences, Sichuan University, Chengdu, China; ^3^University of Chinese Academy of Sciences, Beijing, China

**Keywords:** CYPome, cold, heat, transcriptome, perennial ryegrass, tall fescue

## Abstract

Plant cytochrome P450s are involved in a wide range of biosynthetic reactions that generate various biomolecules, including a variety of defensive compounds. Perennial ryegrass (*Lolium perenne*) and tall fescue (*Festuca arundinacea*) are two major species of turf and forage grasses that usually experience low temperature below −10°C and high temperature over 38°C around the world. In this study, we re-analyzed transcriptome of perennial ryegrass and tall fescue treated with heat and cold stress. Thus, we can evaluate P450 composition in these species and confirm whether P450 genes response to temperature stress. We identified 277 and 319 P450 transcripts with open reading frames larger than 300 bp, respectively. These P450 transcripts were mainly classed in the CYP71, 51, 94, 89, 72, and 734 families. In perennial ryegrass and tall fescue, a total of 66 and 62 P450 transcripts were up-regulated, and 65 and 117 transcripts were down-regulated when subjected to heat stress, respectively. When exposed to cold stress, 60 and 73 transcripts were up-regulated, and 59 and 77 transcripts were down-regulated in perennial ryegrass and tall fescue. Among these differentially expressed transcripts, 64 and 87 of them showed expression level changes that followed the same trend, and these temperature-responsive genes primarily belong to the CYP71, 72 and 99 families. Besides, heat and cold stress altered phenylalanine and brassinosteroid involved P450 transcripts in perennial ryegrass and tall fescue. P450 transcripts involved in the metabolism of these compounds showed a strong response to heat and/or cold stress, indicating that they likely play important roles in temperature acclimation in these two species. The CYPome provide a genetic base for the future functional studies, as well as genetic studies that may improve stress tolerance for perennial ryegrass and tall fescue to extreme temperature.

## Introduction

Cytochrome P450 (CYP, P450) enzymes are monooxygenases that constitute one of the largest protein superfamilies, and are found across all organisms, from bacteria to human. P450s are classified into families and subfamilies based on sequence homology and phylogenetic relationship (Nelson et al., 1996). Family members share 40% amino acid sequence identity or higher, and plants have 127 P450 families that represent around 1% of the plant protein-coding genes (Nelson and Werck-Reichhart, 2011). Benefited from massive genomic sequencing efforts, an increasing number of plant cytochrome P450 complements (CYPome) have been reported in recently years, and currently there are more than 5100 plant P450 sequences that have been annotated (Nelson and Werck-Reichhart, 2011). For examples, *Arabidopsis thaliana* genome contains a total of 245 P450 genes (Nelson and Werck-Reichhart, 2011), the *Oryza sativa* genome encodes 343 P450s (Nelson et al., 2008), and the *Morus notabilis* genome encodes 174 P450s (Ma et al., 2014). P450 enzymes are involved in a wide range of biosynthetic reactions, and are involved in the generation of biomolecules, including defense compounds such as flavonoids (Schuler, 1996; Chapple, 1998). Flavonoids are secondary metabolites widely distributed in plant (Harborne and Williams, 2000), and are produced in response to environmental stress in plants (Shirley, 1996; Brunetti et al., 2013). In the flavonoid metabolism network, several key enzymes were characterized as P450s, such as cinnamate 4-hydroxylase (C4H, CYP73A4), flavonoid 3′-hydroxylase (F3′H, CYP75B), flavonoid 3′,5′-hydroxylase (F3′5′H, CYP75A) (Bell-Lelong et al., 1997; de Vetten et al., 1999; Ayabe and Akashi, 2006; Tanaka, 2006). Another defense mechanisms that plants employ in response to adverse environments is to alter expression of stress-related genes (Krasensky and Jonak, 2012) and gene that are upstream of the phytohormone network (Horváth et al., 2007; Tuteja, 2007; Colebrook et al., 2014; Haffner et al., 2014). Literatures revealed that several P450s play crucial roles in phytohormone metabolism and signaling (Saito et al., 2004; Morrone et al., 2010; Yan et al., 2016), and biotic and abiotic stresses can regulate the expression of P450 genes (Bell-Lelong et al., 1997; Whitbred and Schuler, 2000; narusaka et al., 2004). Most P450 genes induced by abiotic or biotic stresses have promoters with recognition sites for MYB and MYC, ACGT-core sequence, TGA-box and W-box for WRKY transcription factors (narusaka et al., 2004). While these data suggest that P450 expression would be affected by temperature stress, but P450 expression patterns under cold and heat stresses remain largely unexplored.

Perennial ryegrass (*Lolium perenne*) and tall fescue (*Festuca arundinacea*) are perennial cool-season forage and turf grass species that are commonly found across the world (Sleper, 1985). Perennial ryegrass can grow throughout the year. Due to its strong trampling resistance, wear resistance, excellent forage quality, and the ability to cover ground quickly, perennial ryegrass can be used as pioneer plant. Tall fescue has deeper rooting and higher root biomass, which provides tall fescue with better drought tolerance and higher yield potential than perennial ryegrass (Gilliland et al., 2010; Graiss et al., 2011; Cougnon et al., 2014). Although perennial ryegrass and tall fescue can grow well in cool and/or moist environments, they are still not suited for environments that experience extreme heat and cold (Wehner and Watschke, 1981; Thomas and James, 1993; Hannaway et al., 2005). Genome-wide expression profiling of CYPome of cold and heat treated perennial ryegrass and tall fescue can pave the way for the molecular studies that may inform breeding of these perennial grasses to tolerate more extreme temperatures.

In this study, we re-analyzed the temperature-responsive transcriptomes of perennial ryegrass and tall fescue, and identified two CYPomes with 277 and 319 P450 transcripts. Expression patterns of these P450 transcripts under heat and cold stresses were carefully investigated.The CYPomes provide a genetic basis for research on their functions, and expression patterns suggest that P450s play crucial roles in temperature response in perennial ryegrass and tall fescue.

## Materials and methods

### Plant materials and stress treatment

Seeds of perennial ryegrass (*L. perenne* cv. Yatsyn) and tall fescue (*F. arundinacea* cv. Barlexas) were planted in 60 plastic pots (14 cm diameter) filled with organic loam on March 2014, and grown in a greenhouse setting at 18–25°C in Chengdu, Sichuan Province of China. For each plastic pot, about 1.5 g of seeds were planted. After 1 month, the grass plants were clipped to a height of 8 cm, and then irrigated with 200 mL distilled water every 2 days for 10 days prior to the experiment. The grass plants were transferred to a growth chamber (Cool Daylight, 3070 Lm 85 Lm/W, Philips, Thailand) under a 14/10 h day/night cycle, an irradiance of 200 mol·m^−2^s^−1^ (LI- 6400/XT photometer, Li-Cor Inc., Lincoln, NE, USA), temperature of 26°/18°C during the day/night and a relative humidity of 70%. After 2 weeks, 60 plastic pots of each grass species were divided into three groups and exposed to heat stress (HS) at 40°C, cold stress (CS) at −10°C, or control temperature (22°C) for 10 h. Leaves were randomly collected from at least 100 plants in each group and pooled enough as a sample. Accordingly, six leaf samples were collected (RCK, RHEAT, RCOLD, TCK, THEAT, and TCOLD) and snap-frozen immediately and stored in liquid nitrogen for RNA-Seq analyses.

### RNA extraction and high-throughput RNA-sequencing

For each leaf sample, more than 1 g of the sample was ground into powder in liquid nitrogen. Total RNAs were then extracted using TRIzol® reagent (Invitrogen, Carlsbad, CA, USA), following the manufacturer's instructions. Genomic DNAs were removed using DNase I (Invitrogen, Carlsbad, CA, USA). More than 20 μg total RNA was then submitted to Beijing Genomics Institute (BGI)-Shenzhen, Shenzhen, China (http://www.genomics.cn) for quality control. RNA quality and purity was assessed by analysing the OD_260/230_ ratio, and RNA integrity number (RIN) was assessed by SMA3000 and Agilent 2100 Bioanalyzer. Qualified total RNAs were then used for mRNA purification and fragmented cDNA library construction using Truseq™RNA Sample Prep Kit (Illumina, SanDiego, CA, USA). The fragmented cDNA library was then sequenced on Illumina Hiseq 2000 platform at Beijing Genomics Institute (BGI) (Shenzhen, China). The clean PE reads generated by Illumina platform were deposited in the Sequence Read Archive (SRA) database (http://www.ncbi.nlm.nih.gov/Traces/sra/sra.cgi?) under accession numbers of SRP059410 and SRP059405.

### *De novo* assembly, sequence annotation and expression analyses

The clean reads from tall fescue and perennial ryegrass were de novo assembled separately using Trinity (v2013-02-25) with the default parameters (Grabherr et al., 2011). Length distribution was assessed by common perl scripts to generate N50 number, average length, max length, and contig number during different length intervals. BlastX and BlastN sequence similarity searches were conducted using Blast2GO platform (Conesa et al., 2005; Conesa and Gotz, 2008) against the protein databases including NR, Swiss-Prot, the Kyoto Encyclopedia of Genes and Genomes (KEGG, http://www.kegg.jp/), Cluster of Orthologous Groups of proteins (COG, https://www.ncbi.nlm.nih.gov/COG/) and Gene Ontology (GO, http://www.geneontology.org/).

For each species, reads of each sample were aligned back to the assembly separately using perl scripts in the Trinity package (v2013-02-25) (Grabherr et al., 2011). Aligned read number of each assembled sequence was calculated and presented as digital expression levels. These values were then normalized by RESM-based algorithm using perl scripts in the Trinity package (v2013-02-25) (Grabherr et al., 2011) to obtain FPKM (Fragments Per Kilobase of transcripts per Million mapped fragments) values.

Differentially expressed transcripts (DETs) were identified with a threshold of log2 fold-change (log2FC) ≥ 1, padj ≤ 0.05 by SOAP (v2.21) with default parameters (Li et al., 2009). Functional enrichment analyses were performed by a hypergeometric test with a threshold of padj ≤ 0.05.

### Identification and classification of cytochrome P450 genes

Candidates for P450 genes were first identified using cytochrome P450 as a key word to search in the de novo assembled transcriptomes. The candidates were then submitted to the NCBI database for protein Blast analyses to verify the annotation. Family and subfamily classification were performed based on the CYP abbreviations of each candidate sequences acquired from the NR, Swiss-Prot, KEGG, COG, and GO database. All P450 transcripts were categorized into 11 clans according to the criteria described by Nelson et al. (1996). Predicted P450 transcripts were aligned using ClustalW (Thompson et al., 2002) and a maximum likelihood tree was constructed using MEGA6.0 (Tamura et al., 2013) and modified by iTOL (Letunic and Bork, 2016).

### Motif analyses and consensus sequence scanning

P450 transcripts were aligned using BlastX (e-value < 0.00001) against protein databases in the priority order of NR, Swiss-Prot, KEGG, and COG. In this case, transcripts aligned to a higher priority database will not be aligned to lower priority database. Proteins with highest ranks in blast results were then analyzed for coding region sequences. Transcripts that could not be aligned to any database were scanned by ESTScan to generate the amino sequence of the predicted coding region (Iseli et al., 1999). These deduced amino sequences were then subjected for motif and consensus sequence identification.

## Results

### Identification and annotation of P450s in perennial ryegrass and tall fescue

To profile the temperature-responsive transcriptome of perennial ryegrass and tall fescue, cold (−10°C) and heat (40°C) stress treated leaves were sampled and submitted to HiSeq 2000 platform for high-throughput RNA-sequencing. A total of 73,125 and 97,565 contigs were *de novo* assembled for perennial ryegrass and tall fescue, respectively (Wang et al., 2016). Functional annotation of the two transcriptomes was performed using Blast2GO (Conesa et al., 2005; Conesa and Gotz, 2008), and we focused on P450 genes that showed changes in expression upon temperature treatment. As a result, 383 and 453 cytochrome P450 transcripts were identified from perennial ryegrass and tall fescue, respectively, including 277 and 319 P450 transcripts with open reading frame that were longer than 300 bp (Table S1). The corresponding average length was 694.3 and 641.6 bp, and 124 and 172 of the P450 transcripts were longer than 1,000 bp. Annotation analysis showed that 178 LpP450s and 205 FaP450s were categorized as “Second metabolites biosynthesis, transport and catabolism” COG category (Figure S1). In order to further understand the biological functions of the P450 transcripts, KEGG based annotation were performed. A total of 63 and 57 LpP450s were annotated in the pathway of “Stilbenoid, diarylheptanoid and gingerol biosynthesis” and “Polycyclic aromatic hydrocarbon degradation.” In addition, 57 LpP450s were annotated as belonging to the “Bisphenol degradation,” “Aminobenzoate degradation,” and “Limonene and pinene degradation” pathways. Compared with the global transcriptome, LpP450s were mainly enriched in these pathways described above (Table S2). The KEGG annotation results of FaP450s showed that large number of FaP450s was involved in stilbenoid, diarylheptanoid, gingerol, polycyclic aromatic hydrocarbon, bisphenol aminobenzoate, limonene and pinene metabolism. Gene Ontology (GO) analysis categorized LpP450s and FaP450s as belonging to “binding,” “catalytic activity,” “metabolic process,” “single-organism process,” and “electron carrier activity” GO categories. Cytochrome P450s usually contain three key amino acid motifs located at the C-terminus, including PERF consensus motif (PXRX), K-helix region (XEXXR) and the heme-binding motif defined by the position of cysteine. According to GO annotation results, 186 (67.15%) and 218 (68.34%) P450 transcripts had heme-binding activity in perennial ryegrass and tall fescue, respectively. Using the patmatdb software to search for the other two consensus sequences (Rice et al., 2000; Blankenberg et al., 2007), it was found that a total of 198 and 232 P450s contained PXRX motif, 201 and 239 contained XEXXR in perennial ryegrass and tall fescue, respectively.

### Subfamily classification of P450s

**Figure 1:**
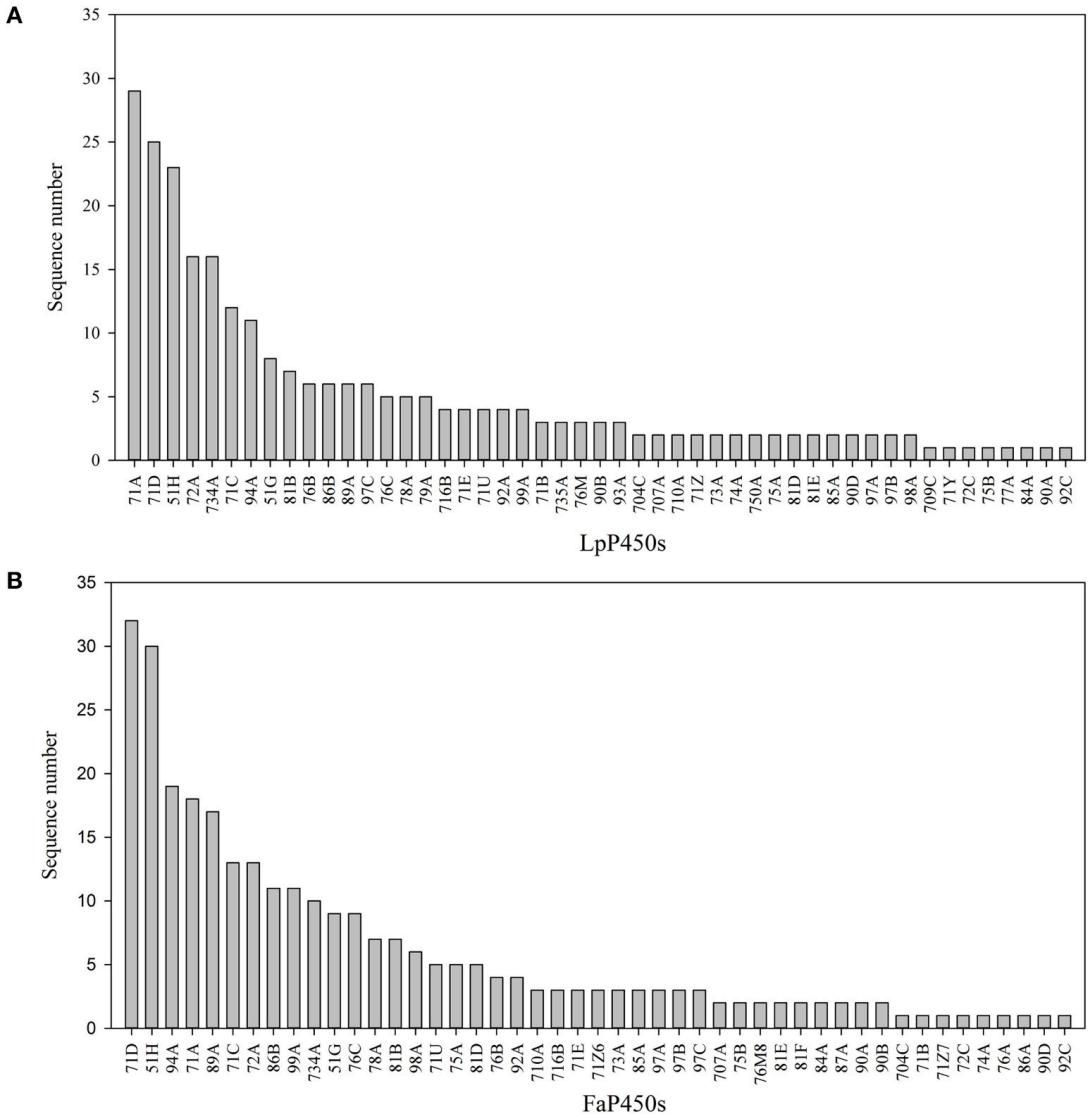
Characteristics of the Predicted P450 Transcripts. Subfamily classification of the P450 transcripts that were identified in perennial ryegrass **(A)** and tall fescue **(B)**.

Different CYP families play different roles in plant metabolism. Perennial ryegrass P450 transcripts were unevenly grouped into 30 families and 49 subfamilies, including 80 transcripts in the CYP71 family, 31 transcripts in the CYP51 family, 17 transcripts in the CYP72 family and 16 transcripts in the CYP734 family (Figure 1, Table 1). Tall fescue P450 transcripts were grouped into 27 families and 47 subfamilies, including 72 transcripts in the CYP71 family, 39 transcripts in the CYP51 family, 19 transcripts in the CYP94 family and 17 transcripts in the CYP89 family (Figure 1, Table 1). Accordingly, CYP71 and CYP51 were the largest families in both species, and the size of the other families varied depending on the species. In tall fescue, 17 CYP89A members were identified, while only six CYP89A were found in perennial ryegrass. CYP79, CYP93, CYP735, CYP750, CYP77, and CYP709 seem to be absent from the tall fescue transcriptome, whereas CYP87, CYP768, and CYP717 were present in tall fescue but absent from perennial ryegrass. These results suggested that these two species may express different cytochrome P450 compositions when exposed to temperature stress.

**Table 1:** Subfamily classification of the predicted P450 transcripts.

**CYP Family**	**Perennial ryegrass**					**Tall fescue**				
	**All**	**Heat-up**	**Heat-down**	**Cold-up**	**Cold-down**	**All**	**Heat-up**	**Heat-down**	**Cold-up**	**Cold-down**
71	80	17	27	17	16	72	8	36	19	25
51	31	16	7	2	9	39	22	6	1	4
72	17	10	1	6	1	14	2	4	2	3
734	16	2	1	6	1	10	0	4	2	1
76	14	0	2	2	5	14	2	8	2	6
81	11	3	0	3	4	16	1	8	1	7
94	11	1	2	0	2	19	1	6	9	1
97	10	0	2	0	0	9	0	2	1	1
86	6	2	2	0	2	12	1	7	1	2
89	6	2	1	2	0	17	5	2	12	0
90	6	0	3	2	4	5	0	4	1	1
78	5	0	3	0	3	7	0	3	0	3
79	5	0	0	2	1	0	0	0	0	0
92	5	0	2	0	1	5	0	4	1	3
99	4	0	2	0	2	11	0	8	0	8
716	4	0	3	2	2	6	0	3	1	2
75	3	0	0	2	0	7	1	0	6	0
93	3	0	1	1	1	0	0	0	0	0
735	3	0	0	1	0	0	0	0	0	0
73	2	2	0	2	0	3	3	0	2	0
74	2	0	0	0	0	1	0	1	0	1
85	2	1	0	0	0	3	0	1	0	1
98	2	1	0	0	0	6	3	0	2	2
704	2	0	0	1	0	1	0	1	0	1
707	2	0	2	0	2	2	1	1	0	1
710	2	2	0	0	0	3	2	0	0	0
750	2	1	1	1	0	0	0	0	0	0
77	1	0	0	1	0	0	0	0	0	0
84	1	0	0	0	0	2	0	2	2	0
709	1	1	0	1	0	0	0	0	0	0
87	0	0	0	0	0	2	0	0	0	0
768	0	0	0	0	0	2	0	0	0	0
717	0	0	0	0	0	1	0	0	0	0

**Figure 2:**
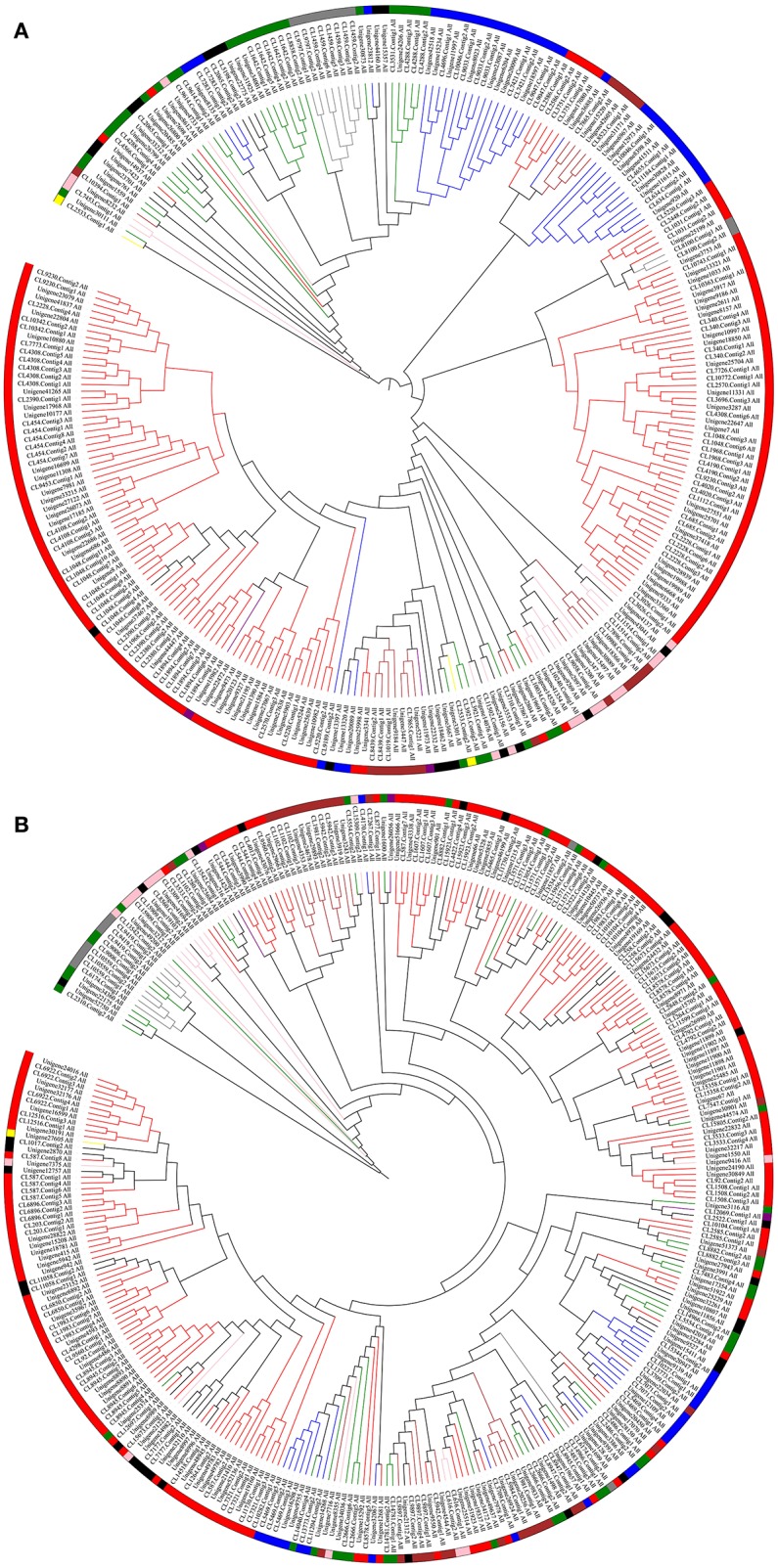
Phylogenetic Tree and Cluster Analyses of the Predicted P450 Transcripts from Perennial Ryegrass and Tall Fescue. Predicted P450 transcripts were aligned using ClustalW (Thompson et al., 2002) and phylogenetic tree was constructed by MEGA6.0 (Tamura et al., 2013) and modified by iTOL (Letunic and Bork, 2016). Different colors indicate different CYP clans. Green, CYP51; red, CYP71; blue, CYP72; yellow, CYP74; pink, CYP85; brown, CYP86; gray, CYP97; purple, CYP710; black, P450 transcripts that cannot be classified into any CYP clan based on the annotation of the trancriptome. **(A)**, perennial ryegrass; **(B)**, tall fescue.

Based on the studies of David Nelson, it is apparent that there are 11 CYP clans in plants, including CYP51, CYP71, CYP72, CYP74, CYP85, CYP86, CYP97, CYP710, CYP711, CYP727, and CYP746 (Nelson et al., 2004; Nelson and Werck-Reichhart, 2011). According to the annotation information, most of the P450 transcripts were clustered into eight clans (CYP51, CYP71, CYP72, CYP74, CYP85, CYP86, CYP97, and CYP710), and CYP711, CYP727 and CYP746 clans appear to be absent from the transcriptomes (Table S1). Besides, the maximum likelihood tree of P450s suggested that several undefined P450 transcripts can be clustered into defined CYP clan. For example, CL1048.Contig4_All (Figure 2A) and CL11058.Contig2_All (Figure 2B) fell into the CYP71 clan.

### Temperature response of the cypomes

**Figure 3:**
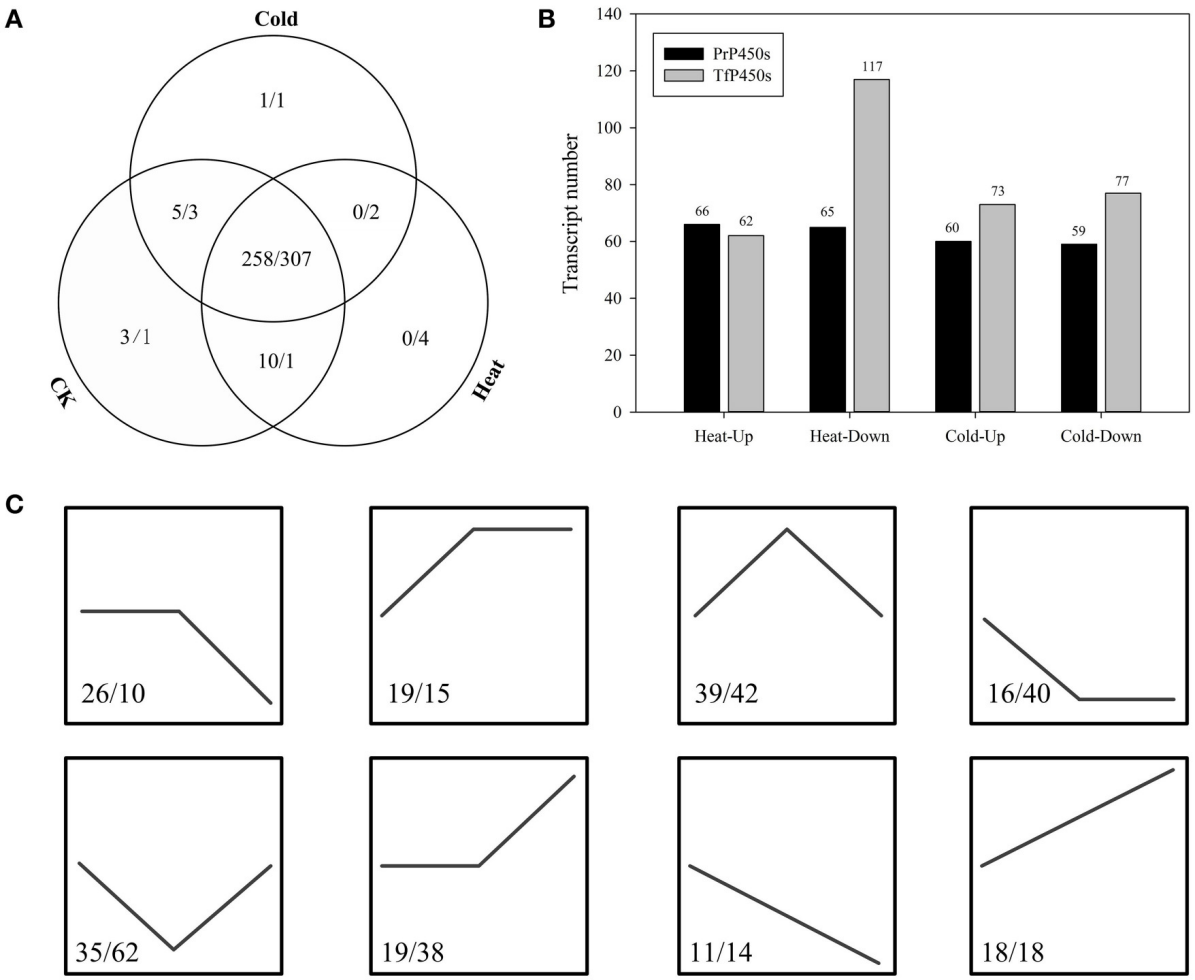
Expression Profiling of the Predicted P450 Transcripts. **(A)** Expression patterns of P450 transcripts that were identified in perennial ryegrass and tall fescue. **(B)** Differential expression of P450 transcripts under heat and cold stress. **(C)** Expression trend of differentially expressed P450 transcripts, where control, heat, and cold were treated as three time points. The numbers before and after the backslash indicate P450 transcript numbers of perennial ryegrass and tall fescue, respectively.

RNA-Seq paired-end (PE) reads of each leaf sample were aligned backed to the corresponding transcriptome separately and the aligned read number was normalized to calculate the FPKM value for each assembled sequence. The results showed that almost all identified P450 transcripts were constitutively expressed in three growth conditions, irrespective of the temperature. In perennial ryegrass, a total of 264, 268, and 274 P450 transcripts expressed in cold, heat and control growth conditions, respectively (Figure 3A). Heat stress significantly up-regulated 66 and down-regulated 65 P450 transcripts, whereas cold stress up-regulated 60 and down-regulated 59 P450 transcripts (Figure 3B). Among the P450 transcripts that were heat significantly up-regulated in the heat treatment, 16 transcripts were members of CYP51H, nine were CYP72A, three were CYP71A, five were CYP71C, and five were CYP71D transcripts(Table S1, A). In contrast, P450 transcripts that were significantly down-regulated in response to heat stress were primarily distributed in the subfamilies of CYP71A (nine) and CYP71D (ten). When exposed to cold stress, six CYP71A, six CYP72A, five CYP71C, six CYP734A, and three CYP71D members were up-regulated, whereas the significantly down-regulated P450 transcripts were primarily distributed in CYP51H (five), CYP71D (six), CYP51G (four), CYP81B (four), and CYP71A (four) subfamilies.

In tall fescue, a total of 313, 314, and 312 P450 transcripts were expressed in cold, heat and control growth conditions, respectively (Figure 3A). Heat stress significantly up-regulated 62 and down-regulated 117 P450 transcripts, and cold stress up-regulated 73 and down-regulated 77 transcripts (Figure 3B). Similar to perennial ryegrass, more than half of CYP51H members (19 of 30) were up-regulated in response to heat stress in tall fescue, whereas more than half of the CYP71D (16 of 32) and CYP71C (7 of 13) transcripts were down-regulated in response to heat (Table S1, B). When exposed to cold stress, 12 of the identified CYP89A members were up-regulated, as well as eight CYP71D and nine CYP94A transcripts. Cold stress resulted in the down-regulation of CYP71D (seven), CYP71C (ten), and CYP99A (eight) members in tall fescue. Among these differentially expressed P450s in perennial ryegrass and tall fescue, 64 and 87 transcripts had the same expression response to cold and heat stress, respectively (Figure 3C). KEGG enrichment analyses demonstrated that these P450 transcripts were enriched in “Stilbenoid, diarylheptanoid, and gingerol biosynthesis,” “Brassinosteroid biosynthesis,” “Polycyclic aromatic hydrocarbon degradation,” “Bisphenol degradation,” “Limonene and pinene degradation” and other second metabolism (such as flavonoid, steroid, and brassinosteroid) related pathways (Table S2).

**Figure 4:**
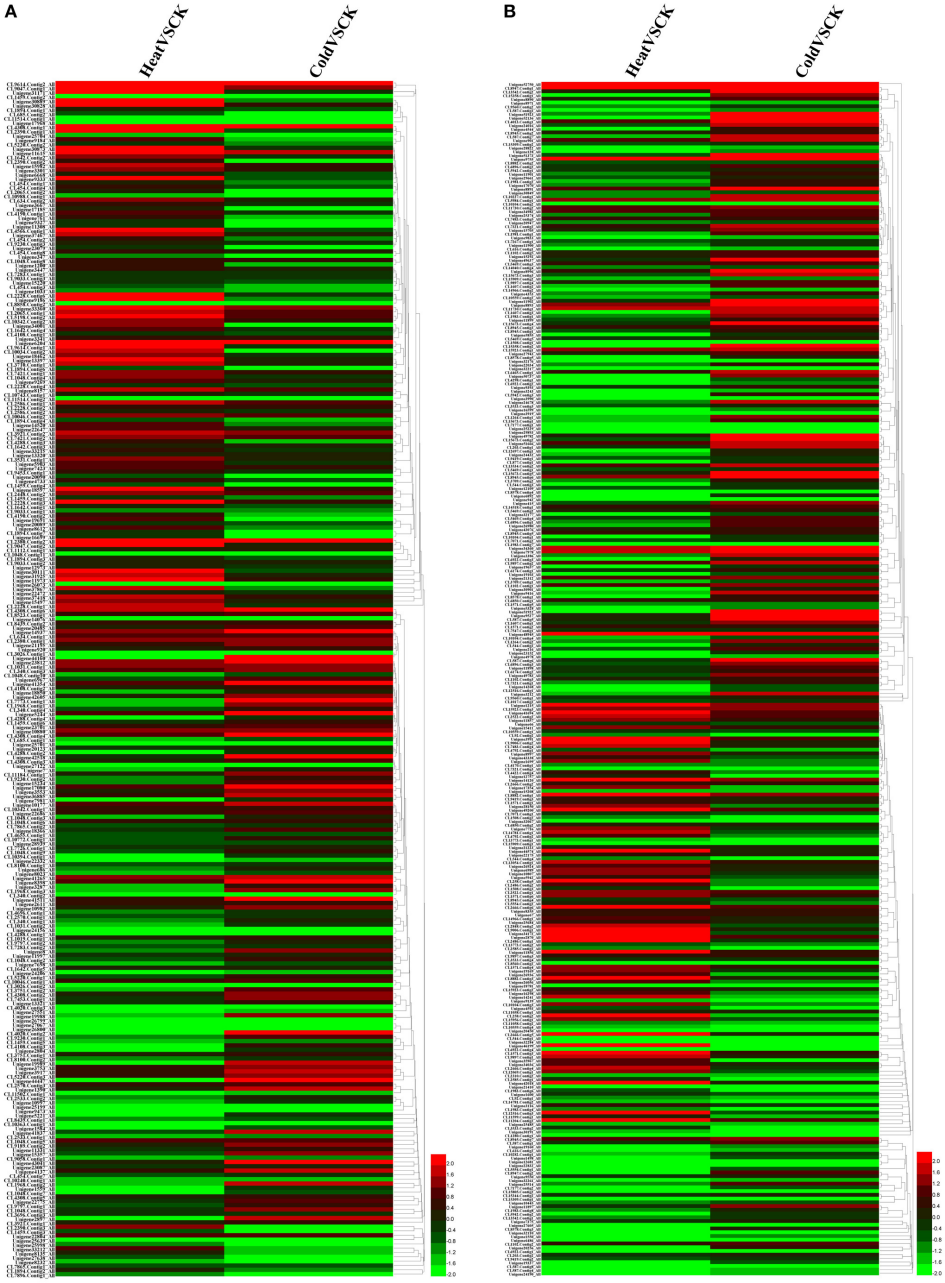
Expression Heatmap of the Predicted P450 Transcripts. Heatmap was drawn according to log2FC values using HemI toolkit (Deng et al., 2014). Log2FC was calculated pairwise basing on the expression level for each P450 transcript. **(A)** Heatmap of P450 transcripts identified in perennial ryegrass. **(B)** Heatmap of P450 transcripts identified in tall fescue.

Without regard to the change fold, more than half of the P450 transcripts suffered an expression change with FDR < 0.05 in response to temperature stresses (Table S1, Figure 4). With this threshold (FDR < 0.05), 184 and 174 P450 transcript had an increased or decreased expression level in perennial ryegrass when exposed to heat and cold stress, respectively. In tall fescue, 242 and 211 P450 transcripts had a changed expression level when exposed to heat and cold stress, respectively. Interestingly, most transcripts showed different changes in expression level when exposed to cold and heat stress (Figure 4).

### Expression patterns of P450 transcripts involved in phenylalanine metabolism

**Figure 5:**
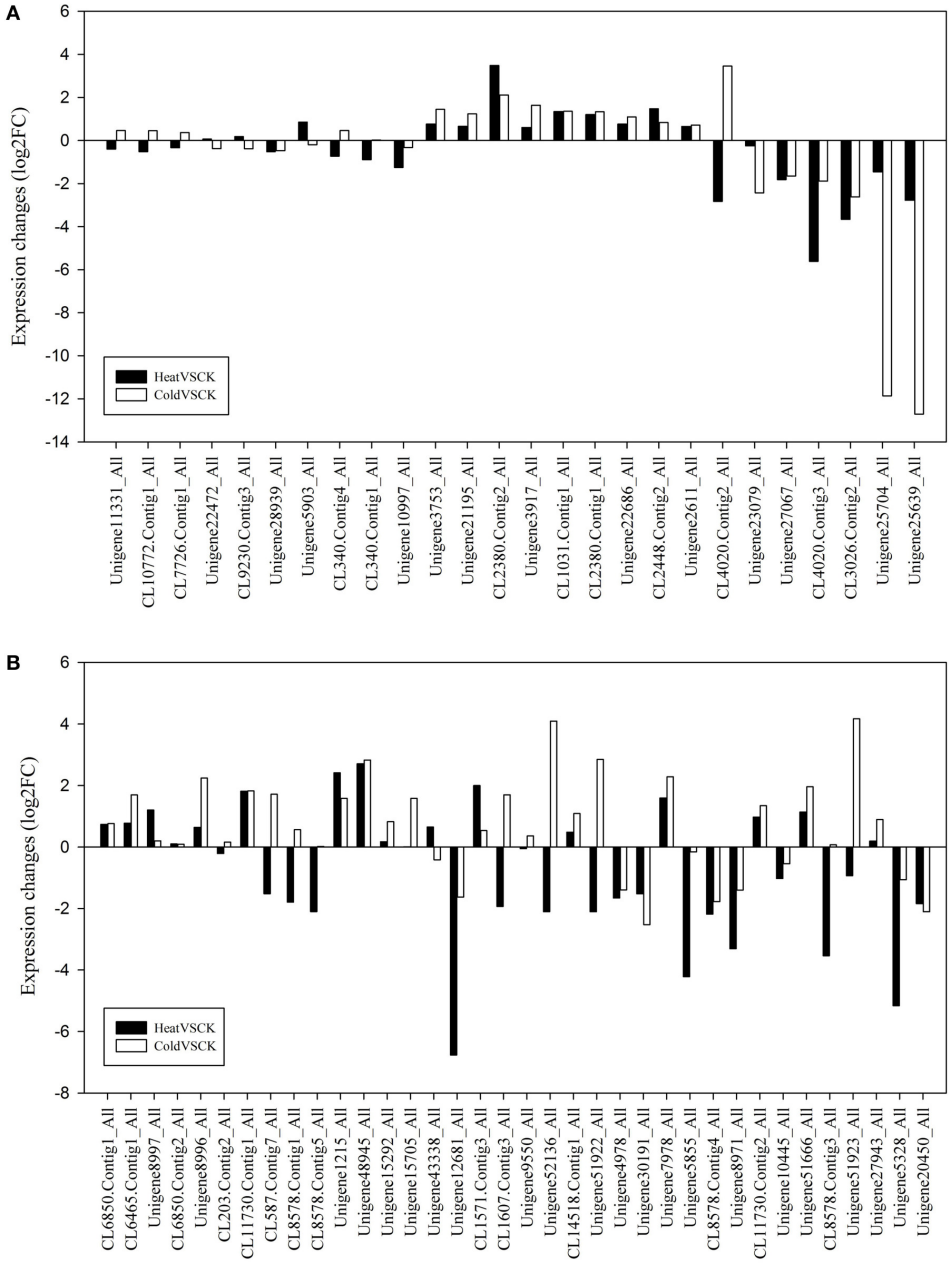
Expression of P450 Transcripts Involved in Flavonoid Metabolism. P450 transcripts involved in flavonoid metabolism in **(A)** perennial ryegrass and **(B)** tall fescue.

Flavonoids are well characterized as defensive compounds and signaling molecules that can withstand a wide range of environmental stresses in plants (Shirley, 1996; Brunetti et al., 2013). In the CYPomes, 26 and 36 flavonoid metabolism related transcripts were identified, including sequences encoding flavonoid 3′,5′-hydroxylase (F3′5′H, CYP75A), flavonoid 3′-hydroxylase (F3′H, CYP75B), cinnamate 4-hydroxylase (C4H, CYP73A4) and 3,9-dihydroxypterocarpan 6a-monooxygenase (CYP93A1) (Table S3). The expression level of most flavonoid related transcripts were affected by heat and/or cold stresses (Figure 5). In perennial ryegrass, expression level of 17 (65.4%) transcripts involved in flavonoid metabolism showed significant change (FDR < 0.05) in expression level when exposed to heat stress, including eight up-regulated and nine down-regulated transcripts. In response to cold stresses, 18 (69.2%) P450 transcript involved in flavonoid metabolism showed significant change in expression level. With this threshold, 24 (66.7%) transcripts involved in flavonoid metabolism showed a change in tall fescue when exposed to heat stress, and 26 transcripts showed change when exposed to cold stress (72.2%). More than half of these P450 transcripts displayed the same change trend (Figure 5). For example, five F3′5′H encoding transcripts were expressed in perennial ryegrass leaves, and two were up-regulated by both heat and cold stresses, and one was down-regulated by heat/cold. Unigene3917_All, the highest expressed F3′5′H transcript, was up-regulated from 105.92FPKM in the control sample to 161.32 FPKM and 329.53 FPKM in heat and cold, respectively. Another F3′5′H encoding transcript, Unigene22686_All, was up-regulated from 2.85 FPKM to 4.83 FPKM and 6.08 FPKM by heat and cold stresses, respectively. In tall fescue, five F3′5′H transcript (Unigene15705_All, Unigene52136_All, Unigene51922_All, Unigene7978_All, Unigene51923_All) were all up-regulated by cold stresses (FDR < 0.05), and Unigene7978_All was up-regulated by heat stresses. C4H is one of the three universal factors involved in flavonoid and lignin biosynthesis (Hahlbrock and Grisebach, 1979). Two (CL2380.Contig1_All, CL2380.Contig2_All) and five (Unigene8996_All, Unigene8997_All, Unigene1215_All, Unigene48945_All, CL6465.Contig1_All) C4H transcripts were identified in perennial ryegrass and tall fescue, respectively. Except for Unigene8997_All, others C4H transcripts were strongly up-regulated (FDR < 0.05) in response to heat and cold stress. Unigene8997_All was also up-regulated by heat (from 3.47 FPKM to 8.01 FPKM) but not by cold stress (4.00 FPKM). These results suggest that heat and cold stresses altered flavonoid metabolism as part of the stress response mechanism.

### Expression patterns of P450 transcript involved in brassinosteroid metabolism

According to KEGG enrichment analyses, transcripts that were differentially expressed in heat and cold treatment samples were also enriched in brassinosteroid (BRs) biosynthesis pathways for both perennial ryegrass and tall fescue (Table S2), indicating the important of this pathway. In perennial ryegrass, 27 LpP450 transcripts were involved in BRs biosynthesis (Table S4). When exposed to heat and cold stresses, 20 and 20 of the 27 P450 transcript involved in BR biosynthesis showed significant change (FDR < 0.05) in expression level, including three steroid 22-alpha-hydroxylase encoding transcripts (DWF4, CYP90B1, EC:1.14.13.-, Unigene8232_All, CL11514.Contig1_All and CL11514.Contig2_All), two steroid 3-oxidase transcripts (D2, CYP90D2, EC:1.14.-.-, CL7453.Contig1_All and Unigene4137_All), one CPD transcript (CYP90A1, Unigene347_All), and two brassinosteroid-6-oxidase 2 transcripts (BR6OX2, CYP85A2, EC:1.14.-.-, Unigene15497_All and Unigene4733_All) (Table 2). Interestingly, most of the transcripts described above were all down-regulated when exposed to heat and cold stresses. In tall fescue, 21 and 17 of the 23 P450 transcripts involved in brassinosteroid biosynthesis showed significant change in expression level when exposed to heat and cold stress. Similarly, most of these P450 transcripts were down-regulated by the changes in temperature (Table 2). Interestingly, PHYB activation tagged suppressor 1 (BAS1, CYP734A1) encoding transcripts that were identified in these two grass species were up-regulated by heat and cold stresses.

**Table 2:** Expression of P450 transcripts involved in brassinosteroid biosynthesis.

	**Gene ID**	**Gene abbreviation**	**CYP family**	**FPKM**			**Log2FC**		**FDR**	
				**CK**	**HEAT**	**COLD**	**HEAT**	**COLD**	**HEAT**	**COLD**
Perennial ryegrass	Unigene347_All	CPD	90A	44.67	21.94	9.99	−1.026	−2.161	0.000	0.000
	Unigene8232_All	DWF4	90B	7.18	2.70	1.25	−1.412	−2.518	0.000	0.000
	CL11514.Contig2_All	DWF4	90B	1.14	0.17	0.00	−2.737	−10.158	0.077	0.035
	CL11514.Contig1_All	DWF4	90B	3.86	0.54	0.00	−2.837	−11.916	0.000	0.000
	Unigene4137_All	D2	90D	3.60	4.94	13.92	0.457	1.950	0.298	0.000
	CL7453.Contig1_All	D2	90D	5.70	4.47	15.34	−0.351	1.428	0.068	0.000
	Unigene15497_All	BR6OX2	85A	2.99	7.69	3.09	1.363	0.048	0.000	0.909
	Unigene4733_All	BR6OX2	85A	3.24	5.56	1.97	0.781	−0.716	0.026	0.170
Tall fescue	CL15909.Contig2_All	CPD	90A	7.44	2.56	4.07	−1.542	−0.872	0.000	0.000
	CL15909.Contig1_All	CPD	90A	12.65	2.89	1.95	−2.132	−2.699	0.000	0.000
	CL13542.Contig2_All	DWF4	90B	0.00	0.00	0.37	N/A	8.547	N/A	0.402
	CL13542.Contig1_All	DWF4	90B	8.39	4.17	7.14	−1.009	−0.234	0.000	0.156
	Unigene3212_All	D2	90D	3.83	1.30	11.73	−1.562	1.615	0.000	0.000
	Unigene7375_All	BR6OX2	85A	4.84	4.58	6.13	−0.080	0.340	0.853	0.414
	CL15309.Contig2_All	BR6OX2	85A	4.71	3.53	4.08	−0.417	−0.205	0.133	0.539
	CL15309.Contig1_All	BR6OX2	85A	1.90	0.40	0.51	−2.254	−1.909	0.013	0.037

## Discussion

### NGS-based cytochrome P450 identification and classification

Large numbers of P450 genes often exist in higher plants due to gene duplication and divergences. Plants depend on P450s for nearly every aspect of their metabolism. Through the recent genome sequencing efforts, P450 genes have been identified in many species, including *A. thaliana* (245), *O. sativa* (332), *Carica papaya* (142), *M. notabilis* (174), *Vitis vinifera* (315), *Populus trichocarpa* (310) and *Physcomitrella patens* (71) (Paquette et al., 2000; Nelson et al., 2004, 2008; Nelson, 2006; Ma et al., 2014). The published transcriptome sequences of perennial ryegrass and tall fescue provide the opportunity to identify P450 genes (Wang et al., 2016). In this study, we used the transcriptomes of perennial ryegrass and tall fescue that were treated with heat and cold stress, and identified a total of 277 and 319 P450 transcripts with 300 bp or larger open reading frames from perennial ryegrass and tall fescue, respectively. There were many fragmented transcripts in the *de novo* assemblies, and transcripts were discarded if they did not have long ORFs. P450 proteins usually contain three key amino acid motifs located at the C-terminus. In mulberry, Ma et al. identified 174 P450 genes and found that these genes contained PERF consensus, K-helix region (XEXXR) and heme-binding motifss (Ma et al., 2014). We subjected the transcripts in our analysis to a similar analysis and found that 404 (186+218) out of 596 deduced sequences contained the heme-binding motif, 430 (198+232) had PERF consensus (PXRX), and 440 (201+239) contained K-helix region (XEXXR). More than 100 sequences did not contain the consensus motifs. We also found that several families appeared to be absent from the perennial ryegrass transcriptome, but were present in tall fescue. CYP79, CYP93 CYP735, CYP750, CYP77, and CYP709 were not found in the tall fescue transcriptome, whereas CYP87, CYP768, and CYP717 were present in tall fescue but absent from perennial ryegrass. These can be a result of the limitation of RNA-Seq method and assembly algorithm, and also the temporal and spatial expression of genes.

David Nelson et al. classified plant P450 genes into 11 CYP clans, including CYP51, CYP71, CYP72, CYP74, CYP85, CYP86, CYP97, CYP710, CYP711, CYP727, and CYP746 (Nelson et al., 2004; Nelson and Werck-Reichhart, 2011). The CYP71 clan now represents more than half of all CYPs in higher plants. Similar to the CYPome of other plants species (Nelson et al., 2008; Ma et al., 2014), it was found that CYP71 is the largest family in perennial ryegrass and tall fescue. Unlike *A. thaliana, O. sativa, C. papaya, M. notabilis, V. vinifera, P. trichocarpa*, and *P. patens* (Paquette et al., 2000; Nelson et al., 2004, 2008; Nelson, 2006; Ma et al., 2014), perennial ryegrass and tall fescue appeared to have more CYP51 members. Previous study suggest that *A. thaliana, C. papaya, M. notabilis, V. vinifera, P. trichocarpa*, and *P. patens* have only one or two CYP51 genes, and *O. sativa* has 10 CYP51 members (Nelson et al., 2008). But perennial ryegrass and tall fescue had a total of 31 and 39 CYP51 transcripts, corresponding to a percentage of 11.2 and 12.2%, respectively. Although the CYP711, CYP727, and CYP746 clans were absent from the transcriptomes, this information still provides a genetic base for research on their functions.

### Cypome in response to heat and cold stresses

Plants cope with adverse environmental changes by altering expression of stress-related genes (Krasensky and Jonak, 2012). Biotic and abiotic stresses can regulate the expression of some P450 genes in responses to stress (Bell-Lelong et al., 1997; Whitbred and Schuler, 2000; narusaka et al., 2004). In this study, we analyzed the expression pattern of CYPs and found that a high number of P450 transcripts showed a change in expression in response to heat and cold stress (Figure 4). In perennial ryegrass, heat stress up-regulated 17 CYP71 transcripts and 16 CYP51 transcripts, corresponding to a percentage of 21.3% (17/80) and 51.3% (16/31), respectively. Heat stress also down-regulated 27 (33.8%) CYP71 and seven (22.6%) CYP51 transcripts. When exposed to cold stresses, 16 (20.0%) CYP71 and nine (29.0%) CYP51 members were down-regulated. In tall fescue, heat stress resulted in the up-regulation of more CYP51 members (22, 56.4%) than perennial ryegrass. These observations may suggest that these two Pooideae members not only possess more CYP51 members than other plant species (Paquette et al., 2000; Nelson et al., 2004, 2008; Nelson, 2006; Ma et al., 2014), but that these CYP51 members may play crucial roles in heat and cold response. Heat stress led to an increase in the expression of most CYP51s, and a decrease in expression of most CYP71s. CYP71 has been characterized as the largest P450 family in *Arabidopsis*, rice and mulberry (Nelson et al., 2008; Ma et al., 2014). According to the classification criteria of CYP families, CYP71 clan now represents more than half of all CYPs in higher plants (Nelson and Werck-Reichhart, 2011). As P450s within the same family usually catalyze subsequent reaction steps or similar reactions on different substrates, the change in expression of many CYP71 transcripts indicated this family may play important roles in temperature response.

In addition, other CYP members were also affected by heat and cold stresses. CYP73A4 (C4H, EC:1.14.13.11) is one of the three key factors involved in flavonoid biosynthesis (Hahlbrock and Grisebach, 1979). The up-regulated expression levels of C4H transcripts suggest that heat or cold stress induces the biosynthesis of the defensive compounds, which is supported by the changes in expression of F3′H (EC: 1.14.13.21) and F3′5′H (EC: 1.14.13.88) transcripts. As F3′H and F3′5′H are involved in catalyzing reactions in the flavonoid biosynthetic pathway (Winkel-Shirley, 2001; Tao et al., 2017), the changes in expression of F3′H and F3′5′H indicate a change of flavonoid composition in response to temperature. Brassinosteroids (BRs) are a class of plant steroidal hormones that promote plant growth and development (Sasse, 2003), and play crucial roles in environmental stress response (Bajguz and Hayat, 2009). BR levels are positively correlated with the tolerance to cold stress (Xia et al., 2009). Responding to temperature stresses, most P450 transcripts involved in BRs biosynthesis were down-regulated in perennial ryegrass and tall fescue. It appears that temperature stress decreased the accumulation of BRs. Considering that the BR content is controlled by a negative feedback mechanism, down-regulation of key biosynthesis genes and up-regulation of degradation related genes may indirectly reflect higher BRs content.

## Conclusion

Temperature-responsive transcriptome was analyzed and two CYPomes were identified from perennial ryegrass and tall fescue, including 277 and 319 P450 transcripts, respectively. These P450 transcripts were classified into CYP51, CYP71, CYP72, CYP74, CYP85, CYP86, CYP97, and CYP710 clans. This two grass species had more CYP51 members when compared with previous reports from other plant species. When exposed to heat and cold stress, expression of a large percentage of CYP71 and CYP51 members were significantly altered, including several P450s involved in flavonoid and brassinosteroid metabolism.

## Author contributions

X-RM and XT conceived this study, designed the experimental plan, analysed data, drafted and revised the manuscript. YW participated in data analyses and manuscript revise. M-XW, Y-FF, YD and PM participated in sample preparing, treating, collecting, total RNA extracting and manuscript revise. All authors read and approved the final manuscript.

### Conflict of interest statement

The authors declare that the research was conducted in the absence of any commercial or financial relationships that could be construed as a potential conflict of interest.

## Supplementary material

Figure S1 COG Classification of the Predicted P450 Transcripts.

Table S1 Annotation, expression profiling and subfamily analyses of P450 transcripts identified in transcriptomes from perennial ryegrass and tall fescue.

Table S2 KEGG enrichment analyses of all identified P450s and differentially expressed P450s in perennial ryegrass and tall fescue.

Table S3 Expression of P450 transcripts involved in phenylalanine metabolism in perennial ryegrass and tall fescue.

Table S4 Expression of P450 transcripts involved in brassinosteroid metabolism in perennial ryegrass and tall fescue.
