# Supplementary figures and images for: Identification and Expression Profile of CYPome in Perennial Ryegrass and Tall Fescue in Response to Temperature Stress
Source: Front Plant Sci. 2017 Nov 20;8:1519. doi: 10.3389/fpls.2017.01519 (PMC5702011; doi:10.3389/fpls.2017.01519)

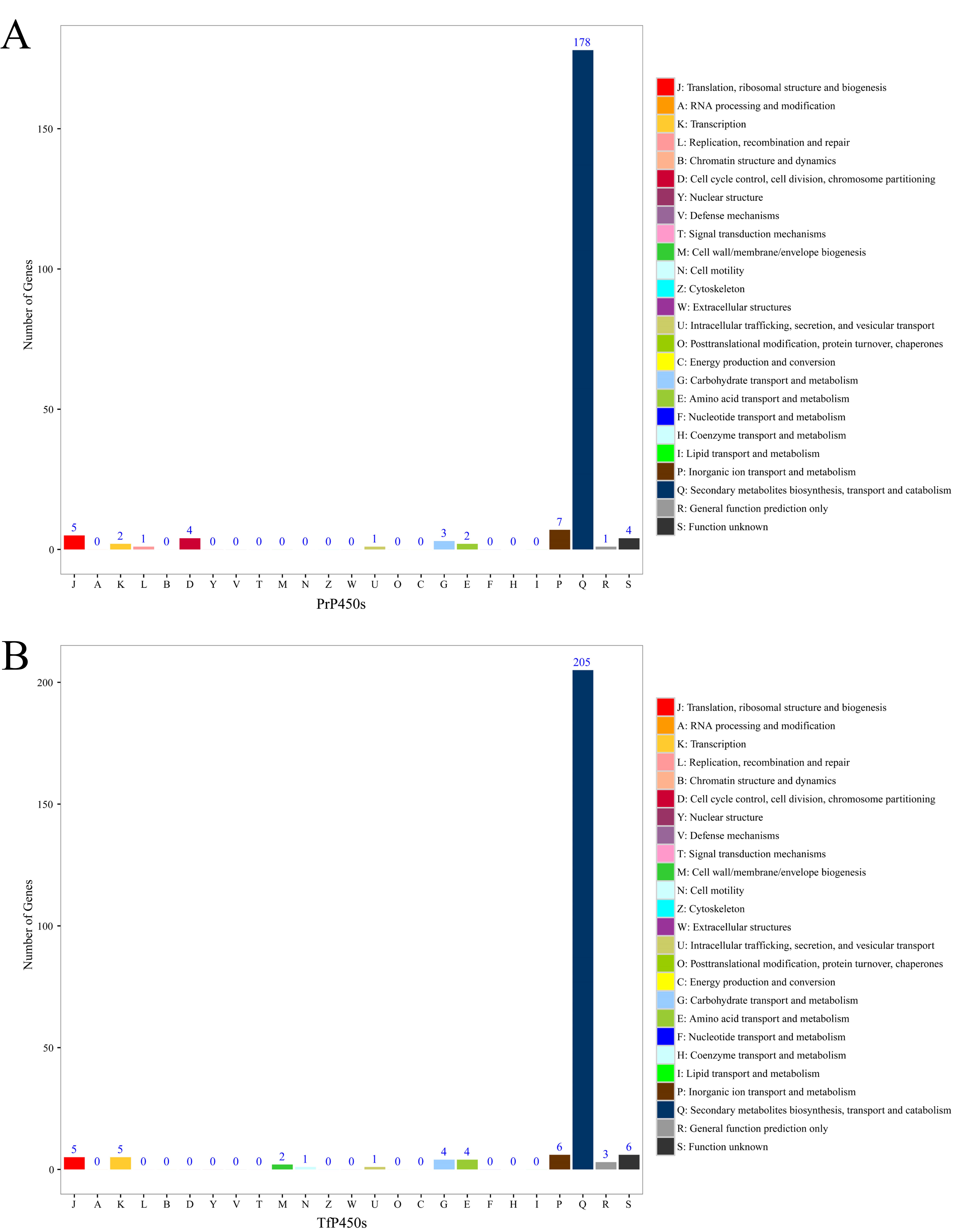

Supplement: Figure S1 — COG Classification of the Predicted P450 Transcripts. [file Image1.JPEG]
